# 172 Costs and Outcomes of Pre-Operative Staphylococcus aureus Screening and Decolonization

**DOI:** 10.1017/ash.2026.10723

**Published:** 2026-06-23

**Authors:** Brandon Smith

**Affiliations:** 1 EST

## Abstract

Ice machines in healthcare environments have increasingly been recognized as potential reservoirs for opportunistic and healthcare-associated pathogens. This study evaluated 43 ice machines across 9 healthcare facilities in the Louisville Metro area during the first half of 2025. Samples were analyzed at a detection limit of 0.1 CFU/mL for key bacterial and fungal organisms associated with hospital-acquired infections. Results revealed no detection of Legionella pneumophila, but notable prevalence of Pseudomonas aeruginosa (4.7%), non-tuberculosis Mycobacteria (NTM) (69.8%), including Mycobacterium chimaera (11.6%), Candida tropicalis (4.7%), Candida glabrata (2.3%), and Acinetobacter baumannii (39.5%), with carbapenem-resistant A. baumannii (CRAB) present in 64.7% of positive A. baumannii sites. Findings highlight substantial environmental contamination in ice machines and reinforce the need for routine monitoring and infection-control strategies. Further, data suggests that healthcare facilities should be aware that more commonly assessed indicator organisms (such as Legionella pneumophila and Pseudomonas aeruginosa) may not provide accurate assessment of the overall microbiological control of these water systems.

